# Atezolizumab‐Induced Immune‐Related Pneumonia on Rounded Atelectasis

**DOI:** 10.1111/crj.70008

**Published:** 2024-09-03

**Authors:** Satoru Yanagisawa, Takaya Yui, Hiroki Takechi, Satoshi Wasamoto

**Affiliations:** ^1^ Department of Respiratory Medicine Saku Central Hospital Advanced Care Center Nagano Japan

**Keywords:** asbestos exposure, atezolizumab, immune‐related adverse events, rounded atelectasis, small‐cell lung cancer

Dear editor:

An 82‐year‐old man with a heavy smoking history (35 pack‐years) was diagnosed with right upper lung small cell lung cancer (extensive‐disease, cT1cN3M1c: cStage IVB, LYM, OSS, HEP) in June 2023. He was a retired electrician who had been exposed to construction dust and asbestos fibers for decades. Chest computed tomography (CT) revealed partially calcified pleural plaques and posterior left lower lobe rounded atelectasis (RA) with “comet tail sign” [1] (Figure 1A,B). Retrospectively, the RA appeared to remain the same shape and size since 2017. Positron emission tomography revealed ^18^F‐fluorodeoxyglucose uptake in the right upper lobe primary tumor, but not in the pleural plaque or RA (Figure 1C–E). Subsequently, the patient was treated with carboplatin/etoposide plus atezolizumab as first‐line chemotherapy in July 2023. Soon after atezolizumab infusion, he developed a transient fever; thereafter, he gradually complained of worsening dyspnea on exertion, with mild desaturation. On day 9 after chemotherapy induction, chest CT showed a new‐onset consolidative shadow on the left lower lung that appeared around the preexisting RA (Figure 2A,B). The laboratory test results, including infectious serology and culture results, were unremarkable. Additional inflammatory serologies (antinuclear and antineutrophil cytoplasmic antibodies) were negative. Due to hypoxemia, further diagnostic studies, such as bronchoscopy, could not be conducted. We suspected that the lesion was consistent with atezolizumab‐induced interstitial lung disease (immune‐related adverse event [irAE]) and started intravenous prednisolone (40 mg daily). After the initiation of steroid treatment, his hypoxemia and lung shadow were almost completely cleared (Figure 2C,D), which supported the diagnosis of irAE pneumonia in RA. We decided to refrain from atezolizumab treatment and continued carboplatin/etoposide therapy alone without recurrence of irAEs.

**FIGURE 1:**
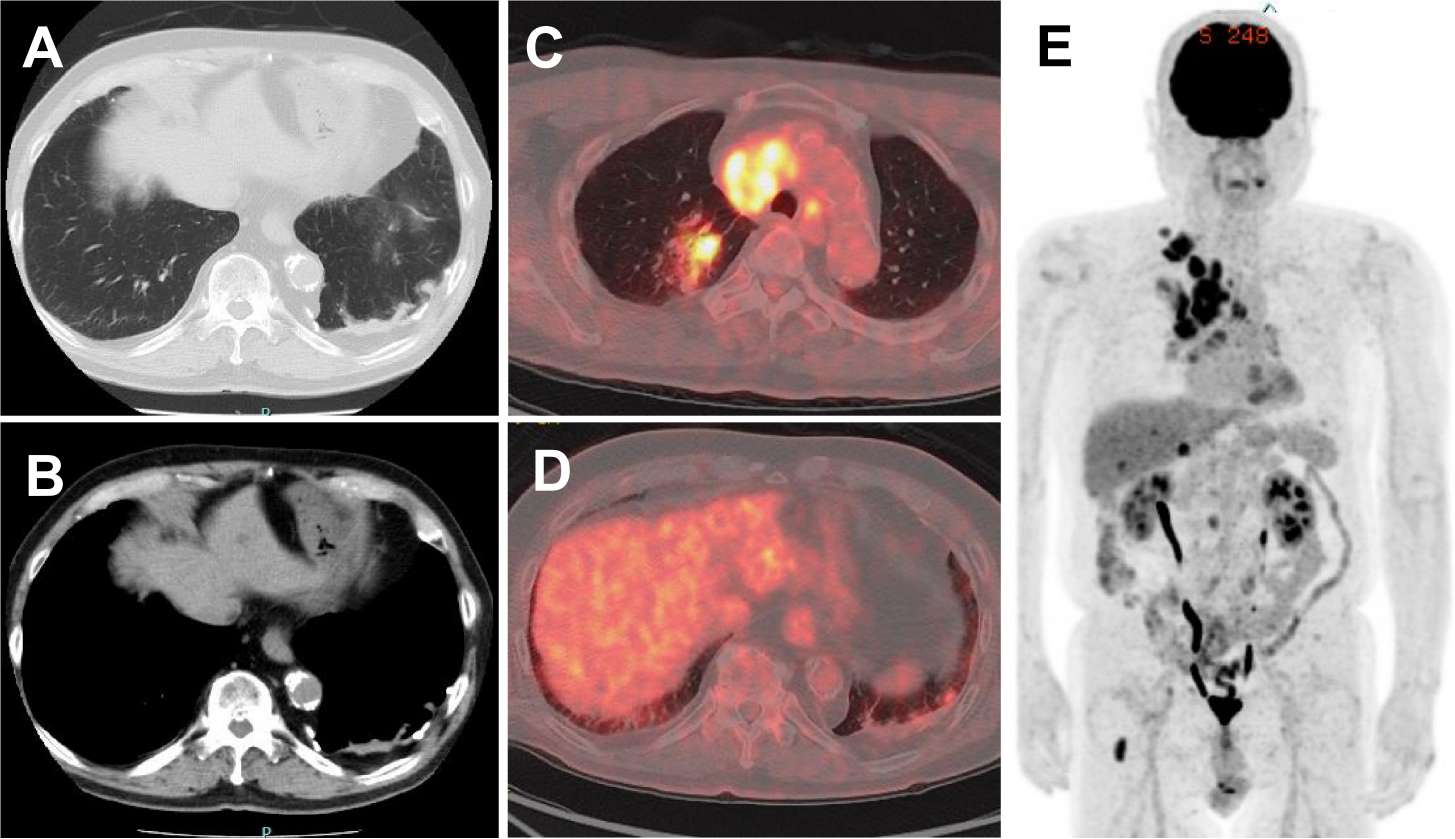
Computed tomography shows a partially calcified pleural plaque and left lower lobe subpleural rounded atelectasis (A and B). Positron emission tomography revealed that ^18^F‐fluorodeoxyglucose uptake was avid only in the right upper lobe nodule and not in the rounded atelectasis (C–E).

**FIGURE 2:**
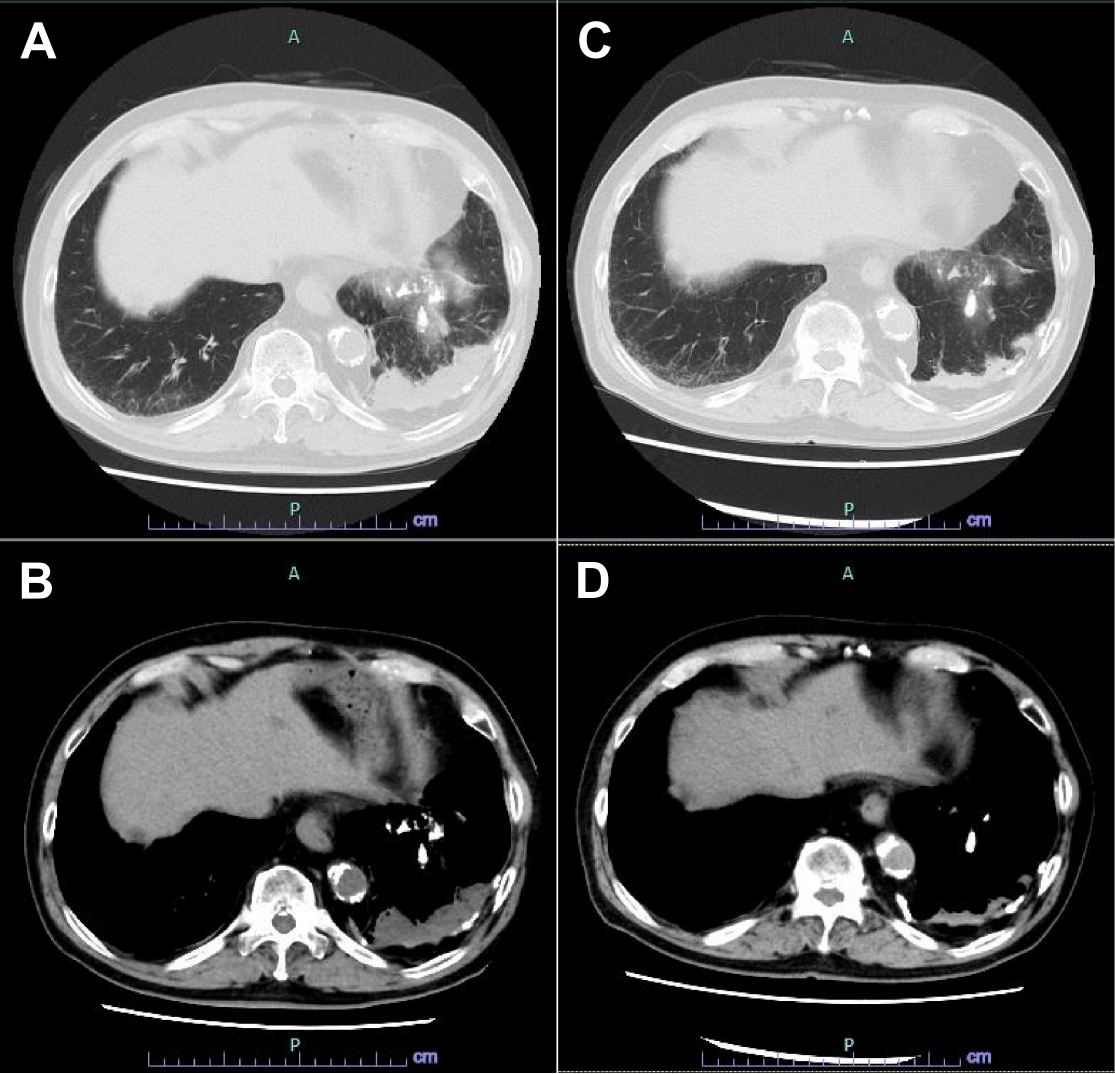
Clinical course of computed tomography findings. An exaggerated consolidative shadow around the rounded atelectasis appeared after atezolizumab treatment (A, B), which cleared after steroid treatment (C, D).

RA [2], also known as “folded lung” or “Blesovsky's syndrome,” is a subtype of lung atelectasis caused by invagination of the redundant visceral pleura [3]. Although most RA are believed to be associated with asbestos lung exposure [4], it is sometimes difficult to differentiate RA from other asbestos exposure‐associated malignant diseases such as lung cancer and malignant pleural mesothelioma [5]. RA usually maintains the same volume and even shrinks on serial scans [4, 5, 6], which supports the benign feature of the lesion and justifies careful follow‐up without intervention. However, there are some reports of RA that gradually enlarge and eventually necessitate surgical biopsy or excision [7]. Although the precise mechanism of RA enlargement is yet to be elucidated, persistent chronic pleural inflammation may be associated. In our case, subpleural consolidation around the RA expanded after the initiation of atezolizumab treatment, and it is possible that the pleural damage around the RA contributed to the occurrence of irAE pneumonia. Sakata et al. reported nivolumab‐induced severe interstitial pneumonia that occurred after talc pleurodesis [8]. They speculated that nivolumab may have exaggerated talc‐induced damage to pleural mesothelial cells, and that chemical inflammation eventually resulted in severe interstitial pneumonia. Although asbestos‐associated RA is usually considered an old inflammatory change, it may be a latent stage with the potential to flare with the use of immune checkpoint inhibitors (ICIs).

In conclusion, this was a case of atezolizumab‐induced irAE pneumonia, which occurred in a patient with RA. As asbestos exposure is associated with RA, it is important to appropriately diagnose drug‐induced pneumonia, which may exaggerate pre‐existing RA. In addition, RA‐associated pleural inflammation may become apparent upon ICIs treatment.

## Author Contributions

Satoru Yanagisawa and Satoshi Wasamoto conceived the study and drafted the manuscript. Hiroki Takechi and Takaya Yui were involved in the discussion and manuscript preparation.

## Consent

All authors reviewed this manuscript and agreed to submit this manuscript.

## Conflicts of Interest

The authors declare no conflicts of interest.

## Data Availability

Data sharing is not applicable to this article as no new data were created or analyzed in this study.
